# Stimulation of platelet P2Y_1_ receptors by different endogenous nucleotides leads to functional selectivity via biased signalling

**DOI:** 10.1111/bph.16039

**Published:** 2023-02-13

**Authors:** Kate L. Arkless, Dingxin Pan, Manu Shankar‐Hari, Richard T. Amison, Clive P. Page, Khondaker Miraz Rahman, Simon C. Pitchford

**Affiliations:** ^1^ Sackler Institute of Pulmonary Pharmacology Institute of Pharmaceutical Science, King's College London London UK; ^2^ School of Immunology and Microbial Sciences King's College London London UK; ^3^ Centre for Inflammation Research The University of Edinburgh Edinburgh UK; ^4^ Chemical Biology Group, Institute of Pharmaceutical Science King's College London London UK

**Keywords:** aggregation, biased‐agonist, chemotaxis, P2Y_1_, platelets

## Abstract

**Background and Purpose:**

Platelet function during inflammation is dependent on activation by endogenous nucleotides. Non‐canonical signalling via the P2Y_1_ receptor is important for these non‐thrombotic functions of platelets. However, apart from ADP, the role of other endogenous nucleotides acting as agonists at P2Y_1_ receptors is unknown. This study compared the effects of ADP, Ap3A, NAD^+^, ADP‐ribose, and Up4A on platelet functions contributing to inflammation or haemostasis.

**Experimental Approach:**

Platelets obtained from healthy human volunteers were incubated with ADP, Ap3A, NAD^+^, ADP‐ribose, or Up4A, with aggregation and fibrinogen binding measured (examples of function during haemostasis) or before exposure to fMLP to measure platelet chemotaxis (an inflammatory function). In silico molecular docking of these nucleotides to the binding pocket of P2Y_1_ receptors was then assessed.

**Key Results:**

Platelet aggregation and binding to fibrinogen induced by ADP was not mimicked by NAD^+^, ADP‐ribose, and Up4A. However, these endogenous nucleotides induced P2Y_1_‐dependent platelet chemotaxis, an effect that required RhoA and Rac‐1 activity, but not canonical PLC activity. Analysis of molecular docking of the P2Y_1_ receptor revealed distinct differences of amino acid interactions and depth of fit within the binding pocket for Ap3A, NAD^+^, ADP‐ribose, or Up4A compared with ADP.

**Conclusion and Implications:**

Platelet function (aggregation vs motility) can be differentially modulated by biased‐agonist activation of P2Y_1_ receptors. This may be due to the character of the ligand‐binding pocket interaction. This has implications for future therapeutic strategies aimed to suppress platelet activation during inflammation without affecting haemostasis as is the requirement of current ant‐platelet drugs.

**LINKED ARTICLES:**

This article is part of a themed issue on Platelet purinergic receptor and non‐thrombotic disease. To view the other articles in this section visit http://onlinelibrary.wiley.com/doi/10.1111/bph.v181.4/issuetoc

## Graphical abstract

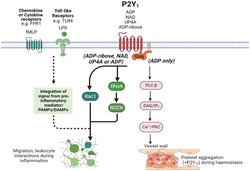

**Abbreviations**
Ap3A: P3‐(5′‐adenosyl) triphosphate
BALF: bronchoalveolar lavage fluid
fMLP: *N*‐formylmethionyl‐leucyl‐phenylalanine
FPR1: formyl peptide receptor 1
GOLD: Genetic Optimization for Ligand Docking software
PPP: platelet poor plasma
PRP: platelet rich plasma
Rac1: Ras‐related C3 botulinum toxin substrate 1
RhoA: Ras homologue family member A
Rho‐GTPase: Ras homologue guanine triphosphate hydrolase family of enzymes
Up4A: uridine adenosine tetraphosphate

**What is already known**

- Platelets participate in inflammation and are necessary for efficient leukocyte recruitment.
- Platelet P2Y_1_ receptor signalling via Rho‐GTPases is necessary for inflammatory functions, rather than PLC signalling.

**What does this study add**

- NAD+, ADP‐ribose, and Up4A induce platelet chemotaxis (not aggregation) via P2Y_1_ activation of Rho‐GTPases.
- NAD+, ADP‐ribose, and Up4A exhibit unique P2Y_1_ receptor docking features compared with ADP.

**What is the clinical significance**

- Future therapeutic strategies to suppress platelet activation during inflammation should not affect normal haemostasis.
- Functionally selective P2Y_1_ receptor antagonism has potential to control platelet function during inflammation.

## 1 INTRODUCTION

The hypothesis of a ‘dichotomy in platelet activation’ was first introduced by Page (1988). In addition to their requisite role in haemostasis, platelets are also important components of the cellular immune system in host defence and in many inflammatory settings. Platelet activation has been shown as an essential precondition for leukocyte activation and recruitment in models of allergic (Amison et al., 2015; Pan et al., 2015; Pitchford et al., 2003) and non‐allergic (Amison et al., 2017; Kornerup et al., 2010; Pan et al., 2015) lung inflammation, as well as infection (Amison, O'Shaughnessy, et al., 2018; McMorran et al., 2009; Youssefian et al., 2002). In these settings, platelet activation is not associated with classical aggregation or parameters of primary haemostasis (Amison et al., 2015; Cleary et al., 2019; Shah et al., 2021). Furthermore, we and others have identified platelets in the lungs of patients with asthma, and in animal models of allergic inflammation, sterile inflammation, and infection, suggesting that this cell type can also undergo extravascular migration into sites of inflammation, the antithesis to an aggregatory event (Amison, O'Shaughnessy, et al., 2018; Cleary et al., 2020; Lê et al., 2015; Ortiz‐Muñoz et al., 2014; Pitchford et al., 2008; Shah et al., 2021). Aberrant platelet activation in the absence of haemostatic dysfunction and thrombosis has also been reported in inflammatory conditions such as rheumatoid arthritis (Boilard et al., 2010), acute lung injury (ALI) (Grommes et al., 2012; Looney et al., 2009), and sepsis (Clark et al., 2007).

Interestingly, platelet activation in the context of inflammatory function appears distinct from activation involved in haemostasis (aggregation). We have previously provided evidence of alternative signalling events (not required for haemostasis) at the platelet P2Y_1_ receptor that were necessary for platelet‐mediated leukocyte recruitment and were suppressed via antagonism of P2Y_1_ (Amison et al., 2017, 2015). These P2Y_1_‐mediated inflammatory responses appear independent of the canonical phospholipase‐C (PLC) signalling pathway, instead occurring via Rho‐GTPase activity (Amison et al., 2015; Amison, Jamshidi, et al., 2018; Pan et al., 2015), as evidence of an example of biased agonism or ‘functional selectivity’ (Kenakin, 2012).

Although P2Y_1_ receptors have been investigated using the cognate agonist, adenosine diphosphate (ADP), other endogenous nucleotides are also known to activate this receptor. P3‐(5′‐adenosyl) triphosphate (Ap3A) has been shown to increase intracellular calcium in 1321N1 cells, downstream of P2Y_1_ receptor activation (Patel et al., 2001). Like ADP, Ap3A is stored in platelet dense granules and released upon cell activation (Lüthje & Ogilvie, 1983). However, Ap3A itself is unable to directly elicit platelet aggregation in vitro, but can do so indirectly through hydrolysis to ADP via plasma hydrolase activity (Lüthje et al., 1985; Lüthje & Ogilvie, 1984). Furthermore, nicotinamide adenine dinucleotide (NAD^+^ ) causes P2Y_1_ receptor‐mediated neuronal hyperpolarisation in ex vivo murine studies (Hwang et al., 2011; Mutafova‐Yambolieva et al., 2007). Although no studies that we are aware of have measured intracellular NAD^+^ concentrations within platelets, they do express CD38 on their surface, which is able to hydrolyse NAD^+^ to ADP‐ribose (Mutafova‐Yambolieva et al., 2007; Ramaschi et al., 1996). ADP‐ribose is another P2Y_1_ receptor agonist, reported to activate this receptor in rat and human primary pancreatic β‐cells through PLC‐mediated increases in intracellular calcium concentrations (Gustafsson et al., 2011). There is also some evidence to suggest that ADP‐ribose may inhibit platelet aggregation (Del Principe et al., 1986), potentially by acting as a competitive inhibitor for ADP. Additionally, uridine adenosine tetraphosphate (Up4A) is another endogenous P2Y_1_ receptor agonist, found to elicit relaxation in human and murine colon muscle (Durnin et al., 2014), whilst also enhancing vascular contraction in mouse (Zhou et al., 2016) and diabetic rat arteries (Mahdi et al., 2018). Again, the presence of this agonist has not yet been investigated within platelets, but it is released from endothelial cells upon their activation (Jankowski et al., 2005).

Although all of the P2Y_1_ receptor agonists described above have the potential to activate platelets, their effects are incompletely understood. Therefore, the aim of the present study was to evaluate a panel of endogenous P2Y_1_ receptor agonists with respect to platelet activation using haemostatic and inflammatory platelet function assays.

## 2 METHODS

### 2.1 Materials

Acid citrate dextrose (ACD)‐A Vacuette tubes (Cat #455055) were purchased from Greiner Bio‐One (Stonehouse, UK). The purinergic receptor agonists, ADP (Cat #01905), NAD^+^ (Cat #N0632), ADP‐ribose (Cat #A0752), and Ap3A (Cat #D1387), the chemotactic peptide *N*‐formylmethionyl‐leucyl‐phenylalanine (f‐MLP) (Cat #F3506), and prostaglandin E_1_ (PGE_1_) were all purchased from Sigma‐Aldrich (Poole, UK). The purinergic receptor agonist, Up4A (Cat #BLG‐U008‐01), was purchased from Enzo Life Sciences (New York, USA). The HTS Transwell 96‐well plates (3‐μm pore size) (Cat #10077792) and RPMI 1640 cell media with L‐glutamine (Cat #12004997) were purchased from Fisher Scientific (Loughborough, UK). The P2Y_1_ antagonist, MRS2500 (Cat #2159/1), the P2Y_12_ antagonist, AR‐C66096 (Cat #3321/1), the PLC inhibitor (Cat # U73122), the Rac1 inhibitor (Cat # NSC23766), and the Rho‐associated kinases (ROCK) inhibitor (Cat #GSK429286) were purchased from Bio‐Techne (Minneapolis, U.S.A). Phycoerythrin (PE)‐conjugated anti‐human CD42b antibody (Cat #555473, RRID:AB_395865), PE‐conjugated rat anti‐mouse CD41 antibody (Cat #558040, RRID:AB_397004), PE‐conjugated rat IgG (Cat #553930, RRID:AB_479719), and fluorescein isothiocyanate (FITC)‐conjugated mouse IgG (Cat #555909, RRID:AB_396216) were obtained from BD Biosciences (Franklin Lakes, USA). FITC‐conjugated anti‐human CD62P (P‐selectin) antibody (Cat #304904, RRID:AB_314476) and PE‐conjugated mouse IgG (Cat #400214, RRID:AB_2800438) were from Biolegend (San Diego, USA). Flow‐Count Fluorospheres (beads, Cat #7547053) and Fibrinogen conjugated Alexa Fluor^488^ (Cat #F13191) were from Beckman Coulter (Indianapolis, U.S.A).

### 2.2 Human platelet isolation

For all studies, blood was collected in accordance with local ethical approval from King's College London (Research Ethics Committee Reference: 10/H0807/99) and adhered to regulations outlined by the Human Tissue Act 2004 as previously described (Amison, Jamshidi, et al., 2018). Blood was collected using ACD‐A Vacuette tubes from healthy male and female volunteers who had not taken non‐steroidal anti‐inflammatory drugs (NSAIDs) or other anti‐inflammatory drugs in the previous 7 days and who were not prescribed anti‐platelet drugs. Whole blood was centrifuged at 133 x *g* for 20 min at room temperature. An aliquot of the upper platelet‐rich plasma (PRP) layer was then used for aggregation studies (see below). To the remaining PRP, 2.5‐μM prostaglandin E_1_ (PGE_1_ ) was added before centrifuging at 800 x *g* for 10 min at room temperature. Platelet‐poor plasma (PPP) was removed and used for aggregation studies (see below). The platelet pellet was resuspended in RPMI 1640 media, again adding 2.5‐μM PGE_1_ and centrifuging at 800 *g* for 10 min at room temperature. Platelets were then adjusted to a final concentration of 5 × 10^7^ platelets mL^−1^ in RPMI 1640 media using an Improved Neubauer chamber (Hawksley & Sons Ltd, Lancing, UK) for chemotaxis studies (see below).

For fibrinogen binding studies, platelets were isolated via gel‐filtration as previously described (Petito et al., 2018). Briefly, PRP was added to a Sepharose C12‐B column and eluted through using HEPES buffer. Only the cloudy platelet containing media eluted from the base of the column was collected. Platelets were then adjusted to a final concentration of 5 × 10^7^ platelets mL^−1^.

### 2.3 In vitro platelet aggregation

The various endogenous purinergic agonists (ADP, Ap3A, NAD^+^, ADP‐ribose, and Up4A) were investigated for their effects on platelet aggregation quantified by light transmission aggregometry of stimulated PRP at 595 nm at 37°C using a SpectraMax 340PC shaking plate reader (Molecular Devices, San Jose, U.S.A) as previously described (Amison, Jamshidi, et al., 2018). Briefly, PRP was stimulated with vehicle (phosphate buffered saline [PBS]) or individual agonists and immediately loaded onto the plate reader. Vehicle stimulated PPP was also used as a control. Measurements were taken at 15‐s intervals for 16 min under shaking conditions. In some studies, PRP was pre‐incubated with vehicle (PBS) or increasing concentrations of the P2Y_1_‐specific antagonist, MRS2500, or the P2Y_12_ ‐specific antagonist, AR‐C66096, for 10 min at room temperature before stimulation with agonists.

### 2.4 In vitro platelet fibrinogen binding

Gel filtered platelets were treated with 2‐mM CaCl_2_. Fluorescently labelled fibrinogen‐AlexaFluor^488^ was added to each 50‐μL platelet sample at a final concentration of 2 μg·mL^−1^. Samples were then allowed to acclimatise for 10 min before stimulation with increasing concentrations of nucleotides for 30 min at room temperature, in the dark. Following incubation, samples were fixed by adding 500‐μL 1% paraformaldehyde (PFA) in PBS for 10 min at 4°C before centrifugation at 1000 *g* for 5 min at 4°C. Supernatants were discarded and samples resuspended in 400‐μL sheath fluid before recording 50,000 events on a Beckman Coulter FC500 flow cytometer.

### 2.5 In vitro platelet chemotaxis

Inflammatory platelet function downstream of purinergic receptor activation induced by endogenous nucleotide agonists was also investigated through in vitro platelet chemotaxis, as previously described, but with minor amendments (Amison, Jamshidi, et al., 2018). Washed platelets (5 × 10^7^ mL^−1^) were treated with 2‐mM CaCl_2_ before stimulation with vehicle (PBS) or individual agonists for 5 min at room temperature. In some studies, platelets were incubated with antagonists for 10 min at room temperature, prior to agonist stimulation. Platelets (80 μL) were then added to the top insert of the 96‐well Transwell plate, with chemoattractant in the bottom well (0/30 nM fMLP in RPMI 1640 cell media). Following 90‐min incubation at 37°C, media from the bottom chamber was stained with Stromatol (1:0.5) and platelets were quantified using an Improved Neubauer haemocytometer and a Leica DM 2000 LED microscope with an ×40 objective lens.

### 2.6 In vitro platelet P‐selectin expression

PRP was isolated as described above and stimulated with nucleotides or an equal volume of PBS (control) for 15 min at room temperature. In some experiments, MRS2500 was added to PRP 10 min prior to stimulation. Cells were then stained and fixed in 1% PFA for analysis of surface P‐selectin (CD62P) expression on the CD42b^+^ gate. Platelets in platelet‐rich plasma (PRP) were identified on the basis of size and presence of CD42b. The CD62P^+^ baseline of the CD42b^+^ events was set at 2% for the control treatment and compared with the agonist treatment. Samples were then analysed on a Beckman Coulter Cytoflex flow cytometer to record 50,000 events.

### 2.7 In vivo analysis of pulmonary platelet accumulation

All animal experiments were performed in accordance with the Animals (Scientific Procedures) Act 1986 with 2012 amendment, with local ethical approval from King's College London. Animal studies are reported in compliance with the ARRIVE guidelines (Percie du Sert et al., 2020) and with the recommendations made by the *British Journal of Pharmacology* (Lilley et al., 2020). Female, BALB/c mice (7–12 weeks) were sourced from Charles River Laboratories Ltd, housed under standard conditions of 22 ± 2°C with a 12:12 light:dark cycle in cages of four mice. Animals were provided with food and water ad libitum and given environmental enrichment in the form of wood shavings, shredded paper, cardboard mouse houses, and tubing. Mice were administered vehicle (saline), ADP (10 mM), ADP‐ribose (10 mM), NAD^+^ (10 mM), and Up4A (1 mM) intranasally (*i.n*.). Following a 24‐h incubation time, mice were terminally anaesthetised following intraperitoneal (*i.p*.) injection of 0.25‐ to 0.3‐mL 25% weight per volume (w/v) urethane. In order to lavage the lungs, the tracheas of terminally anaesthetised mice were exposed through blunt dissection. A small incision was then made, and a 22‐gauge cannula inserted and secured in place using string. Using a 1‐mL syringe, 3× 0.5‐mL 100‐μM EDTA in saline was gently injected into and withdrawn from the lungs (bronchoalveolar lavage fluid BALF). BALF was then stored on ice; 100‐μL BALF was stained with 1‐μL anti‐CD41α antibody, a platelet‐specific marker, for 30 min in the dark and at room temperature. After incubation, 550‐μL PBS and 50‐μL flow‐count fluorospheres (Beckman Coulter) were added. Samples were then analysed on a Beckman Coulter FC500 or Beckman Coulter Cytoflex flow cytometer (for separate experiment using samples from NAD^+^ treated mice and associated vehicle treated control group), using a known concentration of flow‐count fluorospheres and subtracting isotype values to calculate the concentration of CD41α‐positive events (i.e., platelets) in the BALF.

### 2.8 Molecular docking of ligands with the P2Y_1_ receptor

Molecular docking was performed to generate several distinct binding orientations and binding affinity for each binding mode as previously described (Amison, Jamshidi, et al., 2018). Subsequently, the lowest binding free energy was considered as the most favourable binding mode for the system. AutoDock Smina (Koes et al., 2013; Trott & Olson, 2010), which uses the AutoDock Vina scoring function by default, was used for the blind molecular docking of the ligands to the P2Y_1_ structure (protein databank PDB ID: 4XNW,4XNV) for finding the best binding site by exploring all probable binding cavities of the proteins. Smina was performed with default settings, which samples nine ligand conformations using the Vina docking routine of stochastic sampling. Then, Genetic Optimization for Ligand Docking (GOLD) molecular docking was applied for the docking of ADP, Ap3A, NAD, ADP‐ribose, and Up4A to the Smina‐located best binding site of the P2Y_1_ receptor for performing flexible molecular docking as described elsewhere (Jones et al., 1995, 1997). Based on the fitness function scores and ligand binding positions, the best‐docked poses for the ligands were selected. The GOLD molecular docking procedure was performed by applying the GOLD suite in the CSD Discovery software (Jones et al., 1997). The genetic algorithm (GA) was used in GOLD ligand docking software to examine thoroughly the ligand conformational flexibility along with the partial flexibility of the protein (Nissink et al., 2002). Finally, the 2D ligand–protein interaction map was generated using BIOVIA discovery studio visualiser 2021.

### 2.9 Statistical analysis and experimental design

**FIGURE 1:**
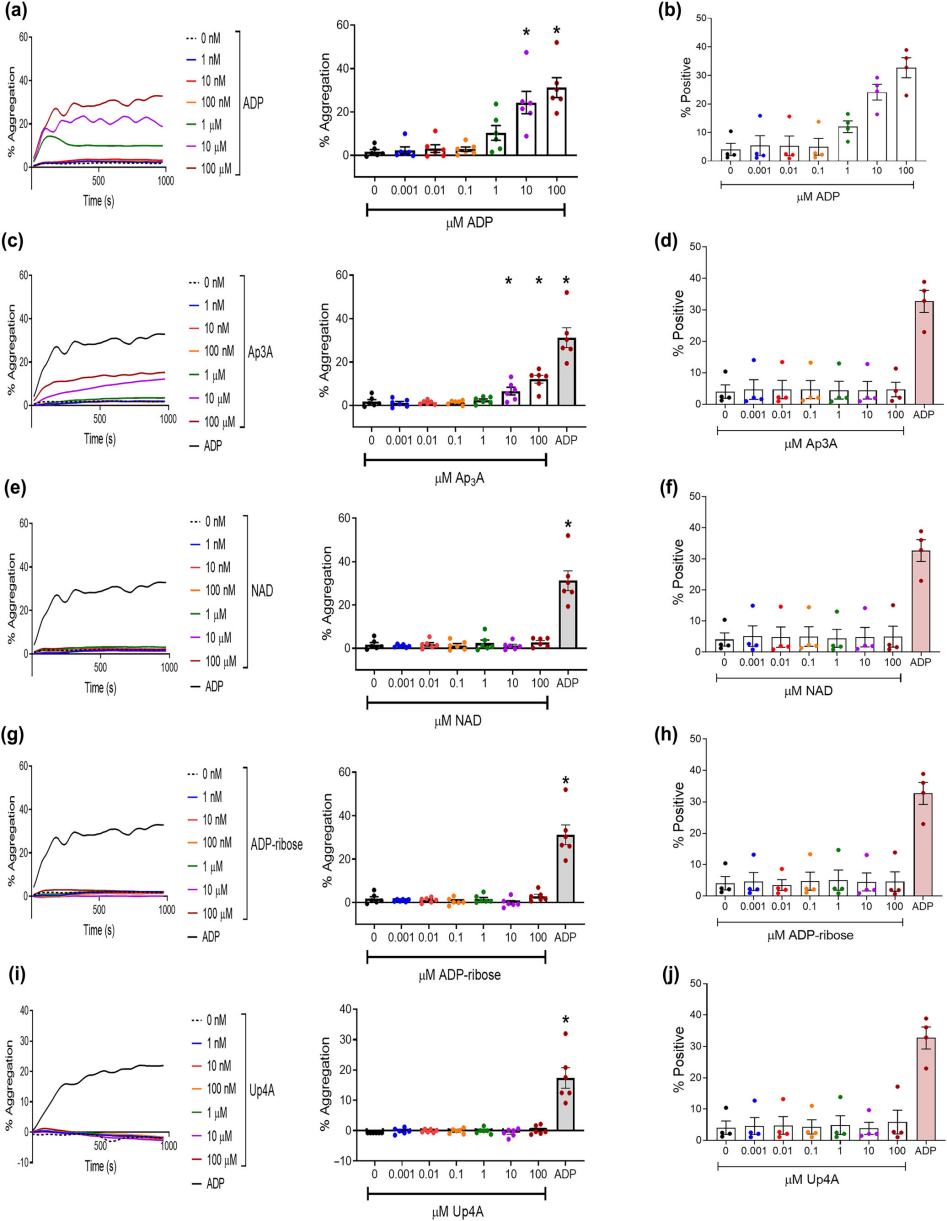
ADP and Ap3A, but not NAD, ADP‐ribose or Up4A, induce platelet aggregation and fibrinogen binding. PRP isolated from healthy human donors was stimulated with increasing concentrations of endogenous P2Y_1_ receptor ligands, and aggregation measured by light transmission aggregometry, with traces showing effect over 16 min, and statistical analysis at 5 min (a, c, e, g, i). In other experiments, fibrinogen‐488 (2 μg·mL^−1^) was added to gel filtered platelets (5 × 10^7^ mL^−1^), and platelets were stimulated with endogenous P2Y_1_ receptor ligands for 30 min before fixation and analysis on a Beckman Coulter FC500 to quantify fibrinogen binding (b, d, f, h, j). (a, b) ADP. (c, d) Ap3A. (e, f) NAD^+^. (g, h) ADP‐ribose. (i, j) Up4A. Data: Mean ± SEM. n = 6 per group (aggregation), and n = 4 per group (fibrinogen binding). One‐way ANOVA with Dunnett's multiple comparisons undertaken on aggregation data only. *P < 0.05 versus negative control left hand column).

Data are expressed as mean ± SEM. Quantification of platelets via microscopy was conducted with the experimenter blinded to the sample identity. All other studies were quantified by machine (plate reader or flow cytometer). Chemotaxis data are normalised to a negative control to give a chemotactic index (CI) of fold mean of control values, due to baseline variations between donors. Groups are of equal size and are indicated in figure legends. Power calculations were undertaken to provide an estimation of the minimum sample size to detect difference between two means, dependent on intra‐group variability of assays based on previous published data (Amison, Jamshidi, et al., 2018; Cleary et al., 2019) or pilot data. In particular, where α‐error (degree of significance) is 0.05, and β‐error (probability of failing to detect a significant difference) is 0.1 (90% power), we calculated a sample requirement for aggregation experiments of n = 5–6 (baseline: 2%, SD: 9.2, expected effect size: 900% increase—this is equivalent to achieving 20% aggregation with ADP from the stated baseline), and chemotaxis studies of n = 6–8 (Positive control group: 1.47 units above baseline of 1.0, SD: 0.48 units, expected effect size to give 50% inhibition of chemotaxis with antagonist incubation). Animal studies were conducted with n = 10 per group, with the group size calculation based on previous published data of LPS‐induced pulmonary platelet recruitment (Control group: 750 units, SD: 245 units, expected effect size 50% increase, Cleary et al., 2019). It should be noted that whilst this study was conducted in female mice, gender differences exist with regard to platelet activation, with reported higher activity in (human) females, although other studies do not reveal a difference (Eshel‐Green et al., 2009; Ranucci et al., 2019; Sabetta et al., 2022). Data were analysed using GraphPad Prism (Version 9), with specific statistical tests indicated in figure legends, where all group sizes were n ≥ 5. Platelet‐fibrinogen binding data was conducted with n = 4 (Figure 1b,d,f,h,j). Statistical analysis was therefore not performed because this exploratory data did not show evidence of changes in platelet adhesion to fibrinogen after incubation with endogenous nucleotides (i.e., the data are ‘negative’), it is therefore confirmatory in nature to the larger dataset of platelet aggregation (Figure 1a,c,e,g,i). In all studies, the group size indicated is the number of independent values, and the statistical analysis was therefore conducted using these independent values. All outliers are included in the data analysis and presentation. In studies where one‐way ANOVA was used (as indicated in figure legends), with multigroup studies with parametric variables, post hoc tests (Tukey's or Dunnett's) were conducted only if *F* in ANOVA achieved *P* value of less than 0.05 and there was no significant variance inhomogeneity. With subsequent post hoc tests, a *P* value of less than 0.05 was considered significant. The data and statistical analysis comply with the recommendations of the *British Journal of Pharmacology* on experimental design and analysis in pharmacology (Curtis et al., 2022).

### 2.10 Nomenclature of targets and ligands

Key protein targets and ligands in this article are hyperlinked to corresponding entries in http://www.guidetopharmacology.org, and are permanently archived in the Concise Guide to PHARMACOLOGY 2021/2022 (Alexander, Christopoulos, et al., 2021; Alexander, Kelly, et al., 2021).

## 3 RESULTS

### 3.1 ADP is the only endogenous P2Y1 receptor agonist that induces platelet aggregation

We have confirmed the well‐established observation that ADP stimulates platelet aggregation via P2Y_1_ and P2Y_12_ receptor stimulation (Figure S1). However, in this study, we have also assessed the ability of other endogenous P2Y receptor agonists to elicit this haemostatic platelet function. PRP was stimulated with increasing concentrations of ADP, Ap3A, NAD^+^, ADP‐ribose, or Up4A. As expected, ADP stimulation led to significant platelet aggregation at both 10 μM and 100 μM, with 100 μM producing the greatest effect (Figure 1a); 100‐μM Ap3A was also found to elicit significant aggregation (*P* < 0.05) n(Figure 1c). However, this is likely due to ADP liberation from Ap3A rather than Ap3A itself, as previously described (Lüthje & Ogilvie, 1984; Lüthje et al., 1985; Figure S2). In contrast, neither NAD^+^, ADP‐ribose nor Up4A were able to elicit in vitro platelet aggregation at any concentration tested (Figure 1e,g,i). As an initial confirmatory measure of in vitro platelet haemostatic function, a flow cytometric assay to measure platelet fibrinogen binding was optimised to understand possible activation of platelets at the single event level, rather than reliance on the optical density measurement provided by measuring platelet aggregation. Fluorescently labelled fibrinogen (fibrinogen‐alexafluor488) was added to gel filtered platelets (5 × 10^7^ platelets mL^−1^) and allowed to acclimatise. Platelets were then stimulated with increasing concentrations of ADP for 30‐min, fixed using 1% PFA and then analysed on a Beckman Coulter FC500 flow cytometer. With similarity to the platelet aggregation data, only ADP stimulation of platelets resulted in increased fibrinogen binding (Figure 1a). Whilst statistical analysis could not be conducted on this exploratory data (n = 4), none of the other endogenous P2YR agonists caused platelet‐fibrinogen binding to occur (Figure 1b,d,f,h,j) and was therefore confirmatory to aggregation data.

Given that platelet fibrinogen binding occurs via an integrin αIIbβ3 conformational change, dependent on P2Y_12_ inhibition of adenylyl cyclase, an enzyme that catalyses the conversion of adenosine triphosphate (ATP) to cyclic adenosine monophosphate (cAMP), it is also suggestive that the endogenous agonists were also not able to cause release of ADP from granular stores, or affect P2Y_12_ activation directly.

### 3.2 ADP and other endogenous P2Y_1_ agonists contribute to platelet chemotaxis as a measure of inflammatory function

In addition to haemostatic function, the panel of endogenous P2Y receptor agonists was also investigated in the context of inflammatory platelet function. The ability of platelets to migrate to sites of inflammation in vivo and to undergo chemotaxis in vitro has been shown by multiple groups (Czapiga et al., 2005; Kraemer et al., 2010; Petito et al., 2018) and has been suggested to require P2Y_1_ stimulation (Amison et al., 2015; Amison, Jamshidi, et al., 2018).

**FIGURE 2:**
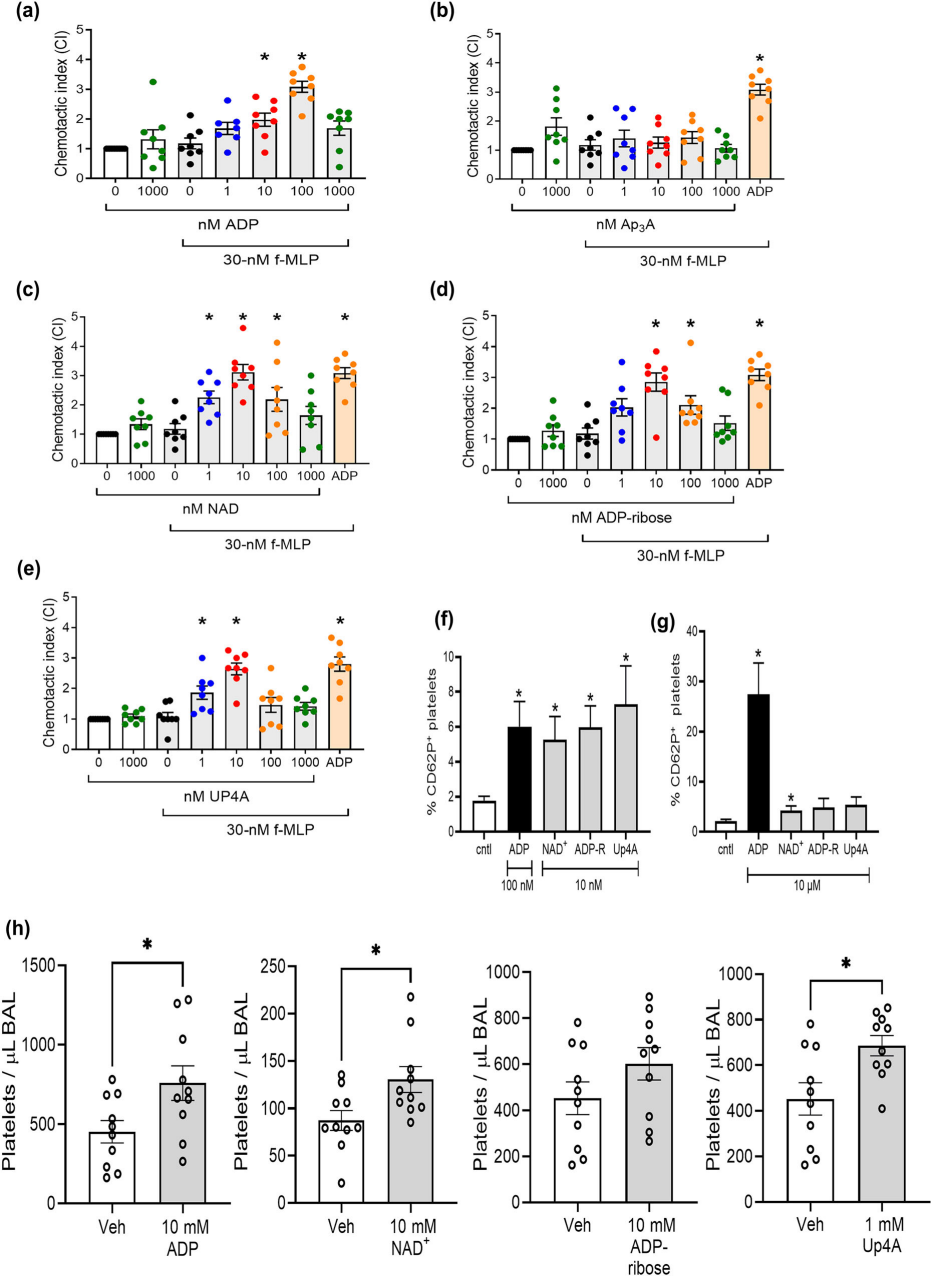
Characterisation of endogenous P2Y_1_ receptor ligands on in vitro platelet chemotaxis, P‐selectin expression, and in vivo lung accumulation. Washed platelets (5 × 10^7^ mL^−1^) in RPMI medium were stimulated with increasing concentrations of endogenous ligands for the P2Y_1_ receptor. Platelets were then added to the top chamber of a 96‐well Transwell plate, with 0/30 nM fMLP in the bottom chamber. After 90 min at 37°C, platelets in the bottom chamber were quantified and normalised to negative controls to give the chemotactic index (CI). (a) ADP. (b) Ap3a. (c) NAD^+^. (d) ADP‐ribose. (e) Up4A. Separately, platelet P‐selectin expression after incubation with ADP, NAD^+^, ADP‐ribose, or Up4A was measured by flow cytometry with concentrations (10 nM or 100 nM, as indicated) that induced chemotaxis (f), or 10 μM (g). In other experiments, BALB/c mice were administered ADP, NAD^+^, ADP‐ribose, or Up4A intranasally, and bronchoalveolar lavage fluid collected 24 h later for platelet enumeration (h). Data: Mean ± SEM. n = 8 per group (a–e), 6 per group (f), 11 per group (g), and 10 per group (h). Analysed by one‐way ANOVA with Dunnett's multiple comparisons. *P < 0.05 versus in the presence of no nucleotide (column C, third from left: a–e), *P < 0.05 compared with vehicle group. (f‐h).

Platelets were stimulated with increasing concentrations of ADP, Ap3A, NAD^+^, ADP‐ribose or Up4A and chemotaxis towards fMLP was measured using a Transwell assay setup. As previously described, platelet stimulation by 100‐nM ADP led to significant platelet chemotaxis towards fMLP in vitro (Figure 2a) (Amison, Jamshidi, et al., 2018). Like ADP, the P2Y receptor agonist, Ap3A, is also present in platelet dense granules and released into the extracellular milieu upon platelet activation and degranulation (Lüthje & Ogilvie, 1983). However, our results indicate that this endogenous P2Y receptor agonist lacked the ability to elicit platelet chemotaxis towards fMLP (Figure 2b). In contrast, NAD^+^ caused significant platelet chemotaxis towards fMLP at 1 nM, 10 nM, and 100 nM (Figure 2c). Similarly, other endogenous P2Y receptor agonists of interest, ADP‐ribose and Up4A, also elicited significant platelet chemotaxis in vitro expressed as bell‐shaped concentration responses. ADP‐ribose caused chemotaxis at 10 nM, and 100 nM, with a peak response at 10 nM (Figure 2d). Finally, Up4A also triggered significant in vitro platelet chemotaxis at concentrations of 1 nM, and 10 nM, with the greatest effect observed at 10 nM (Figure 2e). We next assessed P‐selectin expression on platelets to characterise activation by nucleotides, because P‐selectin expression is also P2Y_1_‐dependent (Anderson et al., 2020), and can be modulated via PLC or Rac1 and RhoA (Akbar et al., 2016, 2007). NAD^+^, ADP‐ribose, and Up4A were able to induce P‐selectin expression at a concentration required for chemotaxis (10 nM), with comparable expression to ADP (100 nM) (Figure 2f). However, this expression did not increase further when platelets were incubated with a higher concentration of nucleotides (10 μM), compared with ADP (10 μM) that causes platelet aggregation (Figure 2g).

Because platelets have been found to migrate to areas of inflammation (for example, the lungs), the pulmonary recruitment of platelets was also investigated after instillation of nucleotides via intranasal delivery (ADP, NAD^+^, ADP‐ribose all 10 mM, Up4A 1 mM), since nucleotide instillation can induce inflammatory cell recruitment (Ferreira et al., 2017). Twenty‐four hours post administration of nucleotides, a significant incidence of platelets was detected in the BAL fluid of mice administered ADP, NAD^+^, and Up4A, but not ADP‐ribose (Figure 2h). These in vitro and in vivo data together suggest the ability of platelets to become activated and motile in response to nucleotides.

### 3.3 Endogenous P2Y nucleotides induced platelet chemotaxis via P2Y1 activation, dependent on non‐canonical RhoA and Rac1 signalling

**FIGURE 3:**
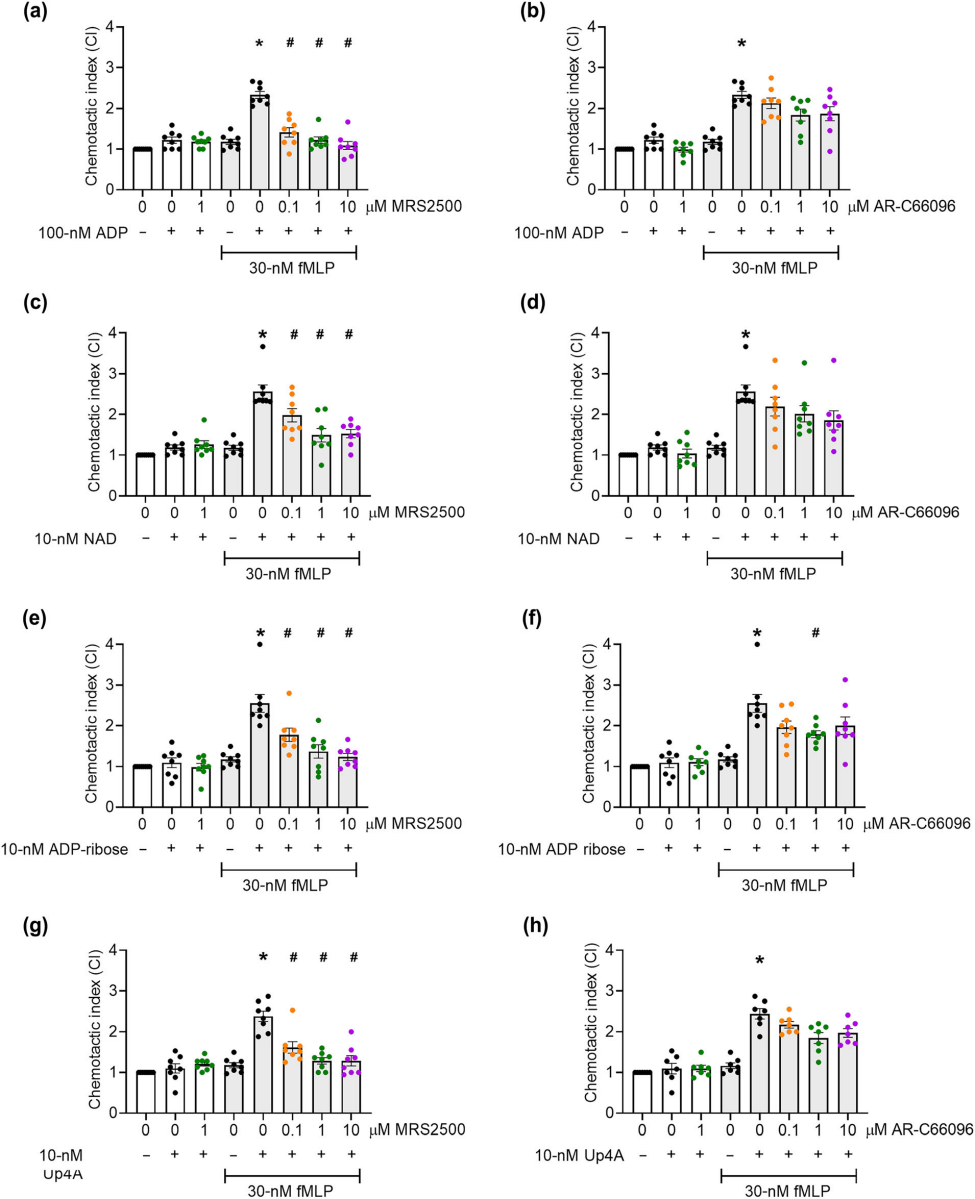
Platelet chemotaxis towards fMLP was induced by ADP, NAD^+^, ADP‐ribose and Up4A via P2Y_1_ receptor. Washed platelets (5 × 10^7^ mL^−1^) in RPMI medium were incubated with specific antagonists for either P2Y_1_ (MRS2500) or P2Y_12_ (AR‐C66096) for 10 min at room temperature. Platelets were then stimulated with increasing concentrations of reported P2Y_1_ receptor ligands and added to the top chamber of a 96‐well Transwell plate, with 0/30 nM fMLP in the bottom chamber. After 90 min at 37°C, platelets in the bottom chamber were quantified and normalised to negative controls to give the chemotactic index (CI). (a, b) ADP‐induced platelet chemotaxis following P2Y_1_ or P2Y_12_ receptor inhibition, respectively. (c, d) NAD. (e, f) ADP‐ribose. (g, h) Up4A. Data: Mean ± SEM. n = 8 per group. One‐way ANOVA with Tukey's multiple comparisons. **P < 0.05 versus in the presence of no nucleotide (column D). ^#^ *P* < 0.05 versus positive control (column 5 from left).

In order to understand the involvement of P2Y receptor activation for endogenous nucleotide induced chemotaxis, we tested this inflammatory function of platelets in the presence of specific P2Y_1_ and P2Y_12_ receptor antagonists. In support of previous studies, we have demonstrated that the P2Y_1_ receptor‐specific antagonist, MRS2500, was able to significantly inhibit in vitro platelet aggregation induced by ADP (Figure 3a); NAD^+^ (Figure 3c); ADP‐ribose (Figure 3e); and Up4A (Figure 3g). However, incubation of platelets with the specific P2Y_12_ antagonist AR‐C66096 had no effect on platelet chemotaxis requiring stimulation by ADP (Figure 3b), NAD^+^ (Figure 3d), or Up4A (Figure 3h), and whilst AR‐C66096 did significantly inhibit ADP‐ribose‐dependent chemotaxis at one concentration (1 μM *P* < 0.05), a considerable level of chemotaxis remained. This single difference makes it difficult to discern a requirement for P2Y_12_ activation (Figure 3f), when compared with the concentration‐dependent antagonism of P2Y_1_ that occurred against all four nucleotides assessed, and reported previously for ADP effects (Amison, Jamshidi, et al., 2018). These data suggest that the endogenous nucleotides, are similar to ADP, in that they stimulate platelet chemotaxis via P2Y_1_ rather than P2Y_12_ receptor activation.

**FIGURE 4:**
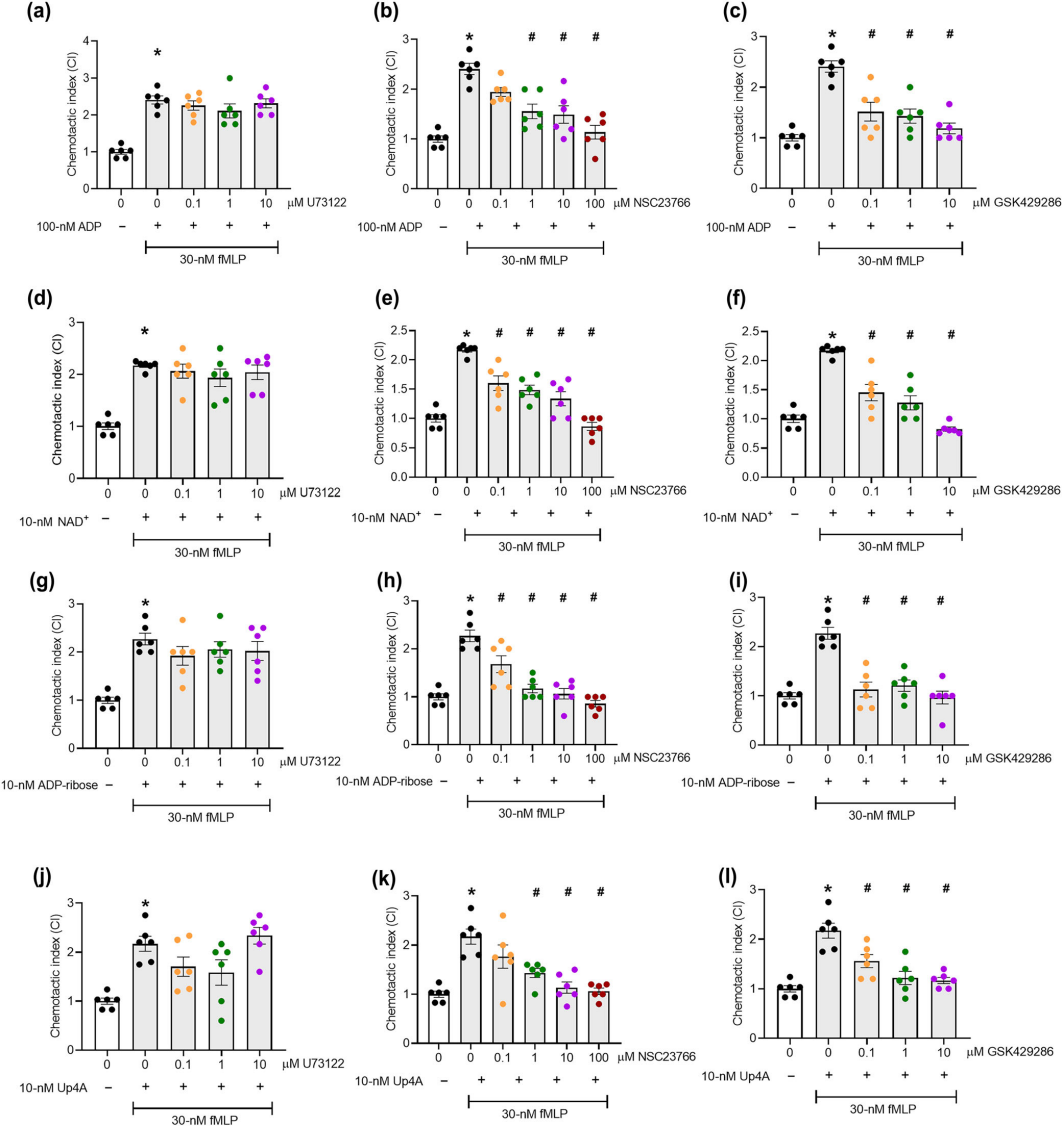
Rac1 and RhoA (ROCK) stimulation are necessary for P2Y_1_ ligand induced platelet chemotaxis. Washed platelets (5 × 10^7^ mL^−1^) were incubated with specific inhibitors of either PLC (U73122), Rac1 (NSC23766), or ROCK (GSK429286) for 10 min at room temperature. Platelets were then stimulated with increasing concentrations of endogenous P2Y_1_ receptor ligands and added to the top chamber of a 96‐well Transwell plate, with 0/30 nM fMLP in the bottom chamber. After 90 min at 37°C, platelets in the bottom chamber were quantified and normalised to negative controls to give the chemotactic index (CI). (a–c) ADP‐induced platelet chemotaxis following inhibition with PLC, Rac1, or ROCK, respectively. (d–f) NAD. (g–i) ADP‐ribose. (j–l) Up4A. Data: Mean ± SEM. n = 6 per group. One‐way ANOVA with Tukey's multiple comparisons. *P < 0.05 versus negative control (column A). ^#^ *P* < 0.05 versus positive control (2^nd^ column).

We next assessed whether NAD^+^, ADP‐ribose, and Up4A P2Y_1_‐induced platelet chemotaxis was dependent on RhoA and Rac1 signalling events, as with ADP‐induced platelet chemotaxis, rather than via activation of the canonical PLC signalling pathway required for Ca^2+^ mobilisation during P2Y_1_ induced platelet aggregation. Platelets were incubated in the presence of 0.1‐, 1‐, and 10‐μM U73122 (PLC inhibitor), NSC23766 (Rac1 inhibitor), or GSK429286 (ROCK inhibitor) before stimulation with endogenous nucleotides, and chemotaxis was induced by fMLP. Incubation with U73122 had no effect on platelet chemotaxis induced by ADP, NAD^+^, ADP‐ribose, or Up4A (Figure 4a,d,g,j). However, chemotaxis was significantly suppressed in the presence of increasing concentrations of NSC23766 (Figure 4b,e,h,k) and GSK429286 (Figure 4c,f,i,l). Thus, the endogenous nucleotides stimulate non‐canonical P2Y_1_ signalling pathways (Rac1, and RhoA) to induce platelet chemotaxis.

### 3.4 In silico molecular docking analyses of endogenous nucleotides to P2Y1 reveal different patterns of interaction compared with the non‐biased agonist ADP

The functional studies described above revealed various endogenous nucleotides were able to activate platelets via P2Y_1_ receptors in a distinct manner compared with the cognate ligand ADP, which is able to activate platelets via canonical signalling pathways (PLC) to elicit aggregation, and alternative signalling pathways (RhoA, Rac1) to elicit motility (chemotaxis). NAD^+^, ADP‐ribose, and Up4A, however, were only able to stimulate platelet chemotaxis, suggesting activation by alternative signalling pathways to aggregation. In order to better understand why these differences occurred between endogenous ligands, we performed a molecular docking analysis to compare their interaction with the P2Y_1_ receptor, with that of ADP. Using ChemPLP score, the binding energies of these endogenous ligands were investigated. The cognate P2Y_1_ receptor agonist, ADP, was found to have a ChemPLP score of 69.72, compared with 67.50 for Ap3A, 69.41 for NAD, 60.76 for ADP‐ribose, and 66.26 for Up4A. Thus, alternative P2Y_1_ receptor ligands appeared to occupy the binding pocket of the P2Y_1_ receptor with a similar affinity to the cognate agonist, ADP, shown through comparable GOLD ChemPLP scores.

**FIGURE 5:**
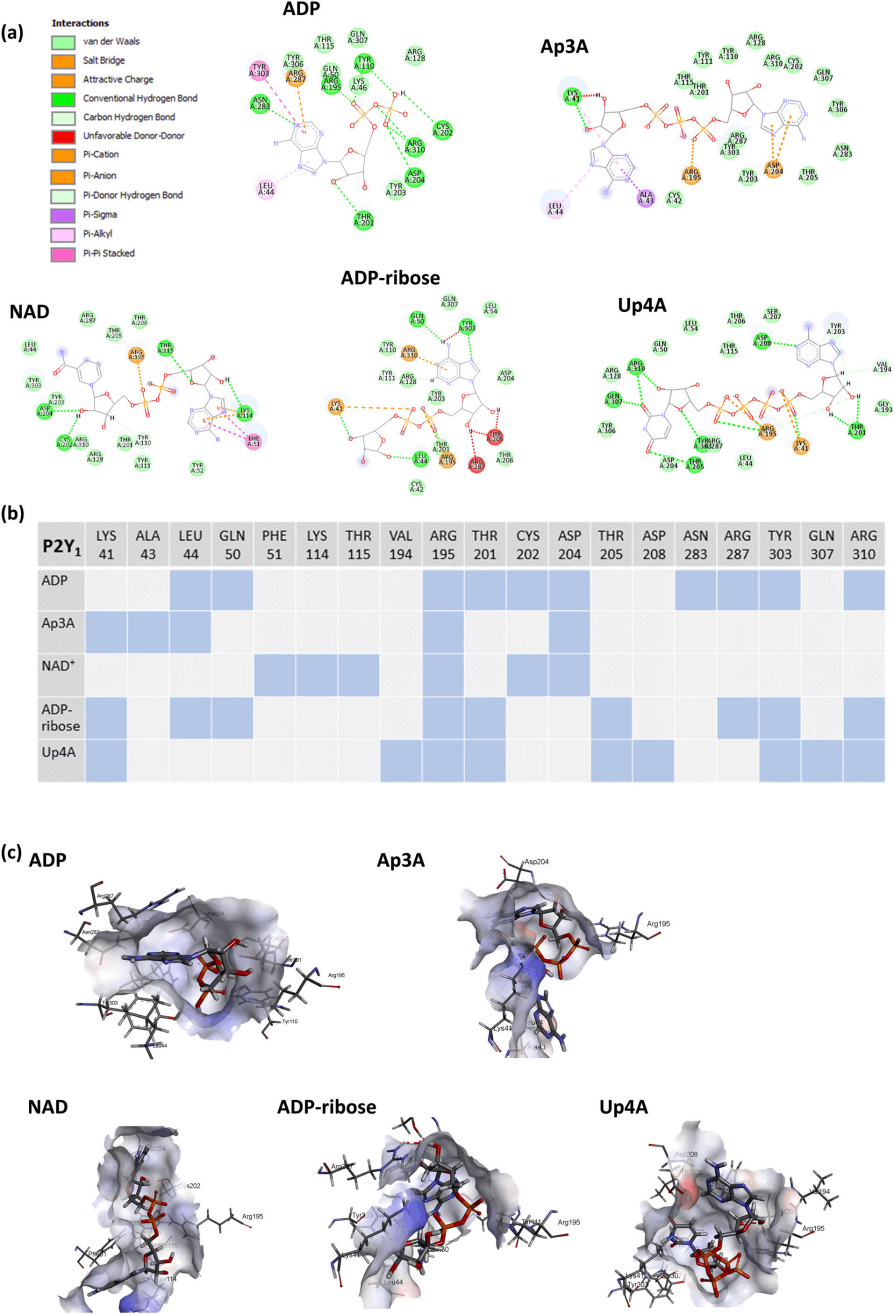
Molecular docking analysis of endogenous P2Y_1_ receptor ligands reveal unique amino acid interactions for non‐cognate ligands compared with ADP. 2D interaction diagrams showing how endogenous ligands bind to amino acids within the P2Y_1_ receptor (a), and comparisons (b), identifying common versus unique contacts. 3D interaction diagram showing the relative position of the endogenous ligands within the P2Y_1_ binding pocket (c).

To understand the differences in the observed in vitro platelet function downstream of P2Y_1_ receptor activation by various ligands, the amino acids involved in these interactions were visualised in silico (Figure 5a) and compared (Figure 5b). Of note, for the P2Y_1_ receptor, ADP was the only ligand found to interact with the polar amino acid, ASN‐283. Only ADP, ADP‐ribose, and UP4A were found to interact with THR‐201, only ADP and NAD interacted with CYS202, and ADP and ADP‐ribose interacted with GLN‐50 and ARG‐287. In comparison, all P2Y_1_ receptor ligands including ADP interacted with the positively charged amino acid (Figure 5b). This suggests that, whilst ADP interacts with about 10 different amino acids within the P2Y_1_ binding site, the endogenous nucleotides (NAD^+^, ADP‐ribose, and Up4A) interact with some of these amino acids and have additional contacts with other amino acids within the binding pocket. Thus, unique patterns of amino acid interaction distinguish the non‐biased P2Y_1_ agonist ADP to nucleotides (NAD^+^, ADP‐ribose, and Up4A) that demonstrated biased agonist properties with respect to P2Y_1_‐dependent platelet function, and this selective interaction with different amino acids within the binding pocket might play an important role in the observed bias for some endogenous ligands. The 3D interaction figures of the ligands with the P2Y_1_ receptor further reinforces this observation, as despite having similar ChemPLP scores, ADP sits deeper into the binding pocket of the P2Y_1_ receptor compared with other ligands which gives rise to differential amino acid interaction (Figure 5c).

## 4 DISCUSSION

Although platelets were once thought of merely as cell fragments involved in aggregation, they are now widely appreciated as critical components of haemostasis, host defence, and inflammation. Evidence also suggests these different functions may be distinctly mediated. Our group have previously shown that inflammatory actions of platelets occur downstream of the platelet P2Y_1_ receptor, in the absence of P2Y_12_ receptor activation (Amison et al., 2017, 2015; Amison, Jamshidi, et al., 2018). With this observation in mind, here we show differential effects of various endogenous nucleotides able to act as agonists at purinergic receptors on haemostatic and inflammatory functions of platelets in vitro and in vivo recruitment.

In this study, we have used in vitro platelet functional assays to understand how platelet P2Y receptors mediate the dichotomy of platelet activation. We have utilised well‐established in vitro assays of platelet aggregation, and fibrinogen binding, using both stimulated PRP or washed platelets to investigate the haemostatic actions of endogenous nucleotides. In addition, we have also investigated the ability of these endogenous nucleotides to induce platelet chemotaxis as an in vitro model of one of the inflammatory actions of platelets given the evidence that they can undergo extravascular migration into various tissues; thus, we also studied platelet accumulation to the lungs in response to intranasal administration of nucleotides in female mice (Cleary et al., 2020; Kraemer et al., 2010; Pitchford et al., 2008; Shah et al., 2021). We have confirmed that platelets can undergo chemotaxis towards the robust chemotactic agent, fMLP as previously described (Amison, Jamshidi, et al., 2018; Czapiga et al., 2005) in the presence of ADP, the cognate purinergic P2Y_1_ and P2Y_12_ receptor agonist. Clearly, directed cell movement requires multiple inputs to control gradient sensing, orientation, and traction (adhesion and de‐adhesion). Using neutrophils as a study platform, Chen and co‐workers reported coordination required a specialised receptor to detect inflammatory or infectious mediators and nucleotide activation to define and regulate the functional response to such inflammatory mediators via Rho‐GTPase activity (Chen et al., 2006, 2010). In particular, FPR1 and P2Y receptors were reported to colocalise as tight spatiotemporal associates at the leading edge of the cell (Chen et al., 2010). This enabled effective nucleotide signalling via autocrine feedback loops, fed by the secretion of ATP from hemichannels activated by FPR1 to amplify the FPR1 response (Chen et al., 2010).

We have also shown that the endogenous nucleotide Ap3A also stimulates in vitro platelet aggregation. However, it has previously been shown that Ap3A itself is incapable of triggering platelet aggregation and that this effect is actually due to inherent hydrolase activity within plasma that converts Ap3A to ADP, which is then able to elicit an aggregatory response (Lüthje et al., 1985; Lüthje & Ogilvie, 1984). Since Ap3A is found in platelet dense granules and is released upon activation (Lüthje & Ogilvie, 1983), it is interesting to speculate its function. It may be that Ap3A acts as a competitive inhibitor to decrease ADP‐induced platelet aggregation or, conversely, may act to sustain the aggregatory signal as it is hydrolysed to ADP. In contrast, none of the other endogenous nucleotides investigated (NAD^+^, ADP‐ribose, and Up4A) were found to exhibit any platelet aggregatory activity.

Interestingly, we have shown for the first time that, along with ADP, the other endogenous P2Y agonists, NAD^+^, ADP‐ribose, and Up4A, are all able to elicit in vitro platelet chemotaxis towards fMLP via activation of P2Y_1_ receptors. When performing a concentration response to increasing ligand concentrations, a bell‐shaped curved was observed. The reasons for this trend are not completely understood, but it could be that, at higher concentrations, the platelet P2Y_1_ receptor undergoes desensitisation and internalisation following ligand stimulation (Baurand et al., 2005; Hardy et al., 2005). Studies of chemotaxis induced by FPR1 receptor stimulation with fMLP on neutrophil‐like NL60 cells have also reported bell‐shaped concentration responses, where subnanomolar concentrations induced chemotaxis, and higher concentrations stimulated other, non‐motile cellular functions (Wang & Ye, 2022). Here, high ligand concentrations might act as a self‐regulatory mechanism to supress further platelet activation, since interaction between the P2Y receptor and adenosine (released or metabolised) signalling pathways (that inhibit both  haemostasis and inflammatory events) has been reported (Layland et al., 2014; Shih et al., 2021). Like ADP, NAD^+^, ADP‐ribose, and Up4A induced chemotaxis via Rac1 and RhoA‐dependent signalling pathways. Thus, their inability to promote platelet aggregation or fibrinogen binding demonstrated an absence of activation of the P2Y_1_ canonical PLC signalling pathway, suggesting that these endogenous nucleotides exhibit biased agonist properties, with functional selectivity of platelet activation involved in inflammation (Kenakin, 2012). It should be noted however, that due to ADP exhibiting a >100‐fold difference in potency across the two functional assays (100‐nM chemotaxis; vs. discernible aggregation at 10 μM), we cannot unequivocally exclude the possibility of system bias (rather than biased agonism) (Smith et al., 2018). However, we note that NAD^+^, ADP‐ribose, and Up4A were able to induce a similar degree of chemotaxis towards fMLP compared with ADP at 10 nM, with no aggregation apparent up to 100 μM. This represents a >10,000 fold difference in concentration with no effect. This leads us to conclude that agonist bias is a probable reason for these functional differences.

In silico analysis to compare the ability of NAD^+^, ADP‐ribose and Up4A to interact with the P2Y_1_ receptor demonstrated that these endogenous ligands interacted with the receptor with similar affinities to ADP, but that their relative position within the binding pocket were slightly different. Furthermore, these experiments showed that the endogenous nucleotides showed interactions with amino acids that differed to those recognised by ADP, providing a potential mechanism by which these biased properties occur.

The relevance of these biased differences in platelet activation induced by different endogenous nucleotides acting on P2Y receptors leading to chemotaxis (i.e., a non‐thrombotic action) without inducing aggregation or fibrinogen binding have not yet been investigated in vivo. However, it is likely that platelet function is influenced by an integrated response to the mixed extracellular milieu of nucleotides (a ‘nucleotide halo’) that can act with distinct properties via the same receptor type, or other purinergic receptors expressed on platelets and activated by distinct nucleotides (for example, ATP and UDP‐glucose), released during trauma, to dictate an inflammatory set of functions, as opposed to haemostatic responses. Greater understanding of this biased agonism pathway may lead to the development of novel pharmacological strategies to target specific platelet functions applicable to inflammation and host defence (Pitchford et al., 2019).

## AUTHOR CONTRIBUTIONS

**Kate Arkless:** Data curation (lead); formal analysis (equal); investigation (equal); methodology (equal); writing—original draft (supporting). **Dingxin (Guest Editor) Pan:** Data curation (supporting); formal analysis (equal); investigation (supporting); methodology (equal); project administration (supporting); writing—review and editing (supporting). **Manu Shankar‐Hari:** Project administration (supporting); supervision (supporting); writing—original draft (supporting). **Richard Amison:** Methodology (equal); project administration (supporting); supervision (supporting); writing—original draft (supporting). **Clive Peter Page:** Funding acquisition (supporting); resources (supporting); supervision (supporting); writing—original draft (supporting); writing—review and editing (supporting). **Khondaker Miraz Rahman:** Conceptualization (supporting); data curation (equal); formal analysis (equal); funding acquisition (equal); investigation (supporting); methodology (equal); resources (equal); supervision (equal); validation (equal); writing—original draft (supporting); writing—review and editing (supporting). **Simon (Guest Editor) Pitchford:** Conceptualization (lead); data curation (equal); formal analysis (equal); funding acquisition (lead); investigation (equal); methodology (equal); project administration (lead); resources (lead); supervision (lead); validation (equal); writing—original draft (lead); writing—review and editing (lead).

## CONFLICT OF INTEREST

No author has a conflict of interest to disclose.

## DECLARATION OF TRANSPARENCY AND SCIENTIFIC RIGOUR

This Declaration acknowledges that this paper adheres to the principles for transparent reporting and scientific rigour of preclinical research as stated in the BJP guidelines for Design and Analysis, and Animal Experimentation, and as recommended by funding agencies, publishers and other organisations engaged with supporting research.

## Supporting information


**Figure S1.** **Mechanic Investigation of ADP‐Induces in vitro Platelet Aggregation**. PRP was incubated with increasing concentrations of antagonist specific for either P2Y_1_ (MRS 2500) or P2Y_12_ (AR‐C6606) for 10 minutes at room temperature. PRP was then stimulated with 100 μM ADP and in vitro platelet aggregation measured. (A) Aggregatory trace of ADP‐induced platelet aggregation following P2Y_1_ inhibition. (B) ADP‐induced platelet aggregation at 5 minutes following P2Y_1_ inhibition. (C‐D) ADP‐induced platelet aggregation following P2Y_12_. Data: Mean (A,C) *P <0.01 versus negative control. ^#^P < 0.05 versus positive control.
**Figure S2. The Effect of Calcium on Ap3A‐Induced Platelet Aggregation, in vitro*.*** Prior to stimulation with 100 μM Ap3A, PRP was incubated with increasing concentrations of CaCl_2_. Platelet aggregation was then measured by light transmission aggregometry, using 100 μM ADP as a positive control. (A) Aggregatory trace over the course of 16 minutes. (**B**) in vitro platelet aggregation at 5 minutes. Data: Mean (A) or Mean ± SEM (B). n = 5 per group. One‐way NOVA with Dunnett's multiple comparisons. *P < 0.05

## Data Availability

The data that support the findings of this study are available from the corresponding author upon reasonable request. Some data may not be made available because of intellectual property rights, privacy or ethical restrictions.
