# Supplementary material for: Stimulation of platelet P2Y1 receptors by different endogenous nucleotides leads to functional selectivity via biased signalling
Source: Br J Pharmacol. 2023 Feb 13;181(4):564–79. doi: 10.1111/bph.16039 (PMC10952403; doi:10.1111/bph.16039)
Supplement: Supplementary file 1 — Figure S1. Mechanic Investigation of ADP‐Induces in vitro Platelet Aggregation. PRP was incubated with increasing concentrations of antagonist specific for either P2Y1 (MRS 2500) or P2Y12 (AR‐C6606) for 10 minutes at room temperature. PRP was then stimulated with 100 μM ADP and in vitro platelet aggregation measured. (A) Aggregatory trace of ADP‐induced platelet aggregation following P2Y1 inhibition. (B) ADP‐induced platelet aggregation at 5 minutes following P2Y1 inhibition. (C‐D) ADP‐induced platelet aggregation following P2Y12. Data: Mean (A,C) *P <0.01 versus negative control. #P < 0.05 versus positive control. Figure S2. The Effect of Calcium on Ap3A‐Induced Platelet Aggregation, in vitro. Prior to stimulation with 100 μM Ap3A, PRP was incubated with increasing concentrations of CaCl2. Platelet aggregation was then measured by light transmission aggregometry, using 100 μM ADP as a positive control. (A) Aggregatory trace over the course of 16 minutes. (B) in vitro platelet aggregation at 5 minutes. Data: Mean (A) or Mean ± SEM (B). n = 5 per group. One‐way NOVA with Dunnett's multiple comparisons. *P < 0.05 [file BPH-181-564-s001.pdf]

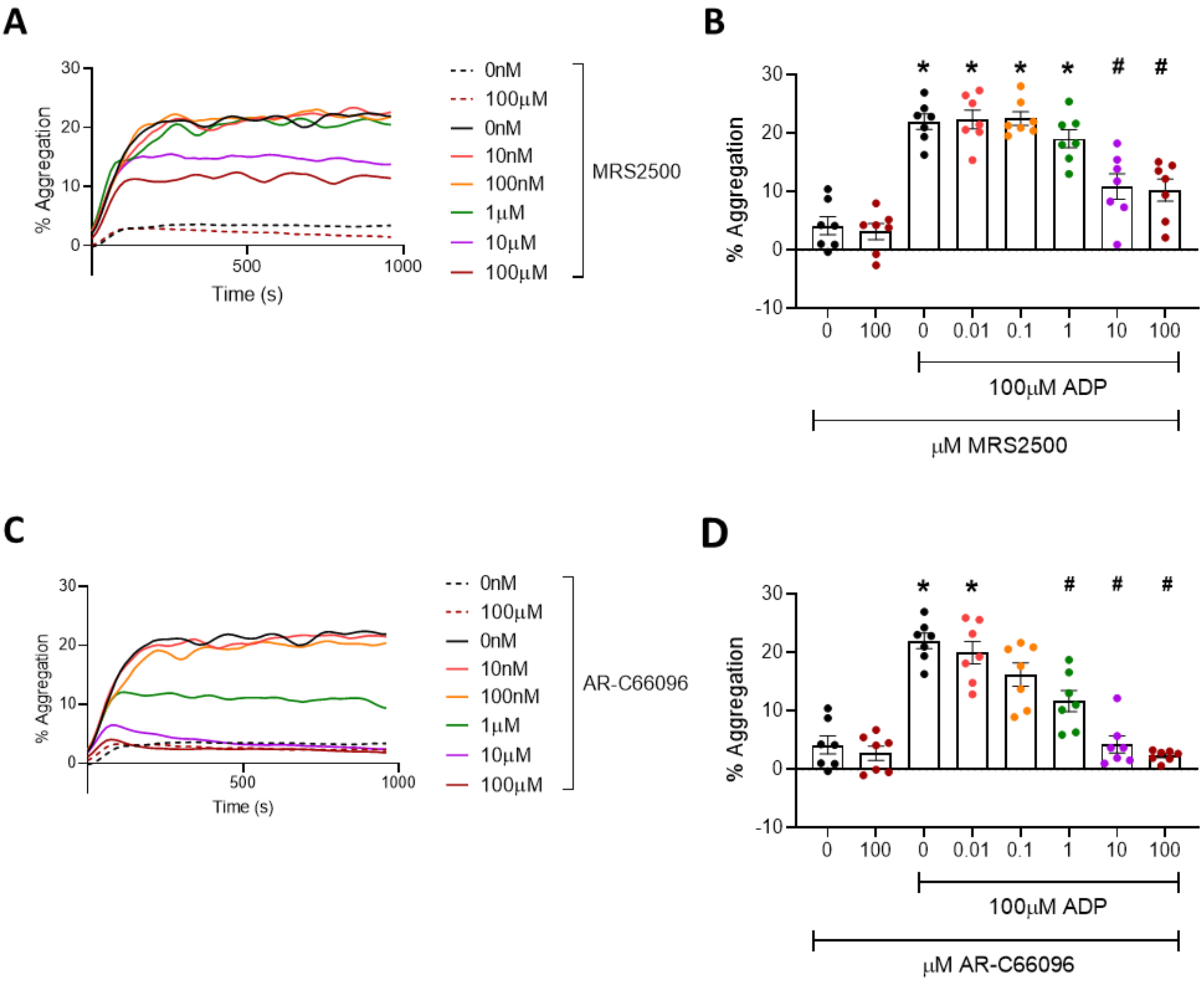

S1. Mechanistic Investigation of ADP-Induced *in vitro* Platelet Aggregation. PRP was incubated with increasing concentrations of antagonists specific for either $P2Y_1$ (MRS 2500) or $P2Y_{12}$ (AR-C6606) for 10 minutes at room temperature. PRP was then stimulated with 100µM ADP and *in vitro* platelet aggregation measured. (**A**) Aggregatory trace of ADP-induced platelet aggregation following $P2Y_1$ inhibition. (**B**) ADP-induced platelet aggregation at 5 minutes following $P2Y_1$ inhibition. (**C-D**) ADP-induced platelet aggregation following inhibition of $P2Y_{12}$. Data: Mean (A,C) or Mean ± SEM (B,D). n = 7 per group. One-way ANOVA with Tukey's multiple comparisons. *P<0.01 versus negative control. $^{\#}$P<0.05 versus positive control.

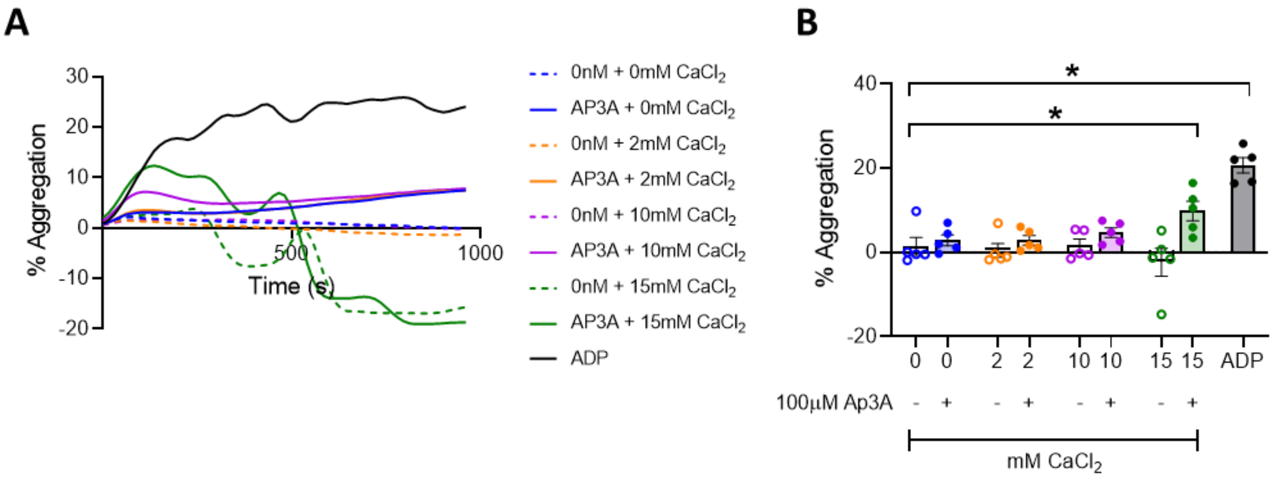

S2. The Effect of Calcium on Ap3A-Induced Platelet Aggregation, *in vitro*. Prior to stimulation with 100µM Ap3A, PRP was incubated with increasing concentrations of $CaCl_2$. Platelet aggregation was then measured by light transmission aggregometry, using 100µM ADP as a positive control. (**A**) Aggregatory trace over the course of 16 minutes. (**B**) *in vitro* platelet aggregation at 5 minutes. Data: Mean (A) or Mean ± SEM (B). n = 5 per group. One-way ANOVA with Dunnett's multiple comparisons. *P<0.05.
